# Abatacept reduces synovial regulatory T-cell expression in patients with psoriatic arthritis

**DOI:** 10.1186/s13075-017-1364-3

**Published:** 2017-07-05

**Authors:** Agnes Szentpetery, Eric Heffernan, Martina Gogarty, Lisa Mellerick, Janet McCormack, Muhammad Haroon, Musaab Elmamoun, Phil Gallagher, Genevieve Kelly, Aurelie Fabre, Brian Kirby, Oliver FitzGerald

**Affiliations:** 1 Department of Rheumatology, St. Vincent’s University Hospital, Dublin, Ireland; 2 Department of Radiology, St. Vincent’s University Hospital, Dublin, Ireland; 3 School of Biochemistry and Immunology, TCD, Dublin, Ireland; 4 Department of Histopathology, St. Vincent’s University Hospital, Dublin, Ireland; 5 Research Pathology, Immunohistochemistry Core Facility, UCD Conway Institute, UCD School of Medicine, Dublin, Ireland; 6 Department of Dermatology, St. Vincent’s University Hospital, Dublin, Ireland

**Keywords:** Abatacept, Psoriatic arthritis, Synovium, Skin, Regulatory T-cell, FOXP3

## Abstract

**Background:**

The aim was to study changes in immunohistochemical expression markers of synovial and skin inflammation, clinical outcomes and magnetic resonance imaging (MRI) scores with abatacept treatment in patients with psoriatic arthritis (PsA).

**Methods:**

Biological-treatment-naïve PsA patients with active disease including synovitis of a knee were enrolled in this single-centre, crossover study. Patients were randomised to receive intravenous abatacept 3 mg/kg of body weight or placebo infusion on day 1, 15 and 29; thereafter abatacept 10 mg/kg of body weight was administered every 28 days for 5 months. Clinical data were collected at each visit. Synovial biopsy of the involved knee was obtained at baseline and 2 and 6 months. MRI of the same knee and skin biopsy was performed prior to arthroscopy.

**Results:**

Fifteen patients were recruited. Significant improvements in the joint-related measures were observed; 90% were European League Against Rheumatism criteria responders and 30% achieved psoriasis area severity index (PASI)50 at 6 months. Reduction in synovitis (*P* = 0.016) and vascularity (*P* = 0.039) macroscopic scores consistent with decrease in total MRI score (*P* = 0.016) were noticed. Abatacept decreased the immunohistological expression of FOXP3+ cells (*P* = 0.027), specifically the expression of CD4+FOXP3+ regulatory T cells (Tregs) (*P* = 0.008) in the synovium over 6 months. There was no significant clinical or immunohistological change in any of the skin measures.

**Conclusion:**

This is the first study assessing synovial and psoriatic skin immunpathological changes following abatacept treatment in PsA. Reduction in Treg expression in the synovium but not in the psoriatic lesion suggests abnormal Treg function in PsA with differential suppressive capacity in the synovium compared to the lesional skin. The results of this study demonstrate that abatacept 10 mg/kg of body weight might be an effective treatment option for joint disease in patients with PsA.

**Trial registration:**

Irish Health Products Regulatory Authority. Trial registration number: CT 900/489/1 – Abatacept (case number: 2077284, EudraCT Number: 2009-017525-19, Protocol number: 77777). Registered on 12 March 2010.

## Background

Psoriatic arthritis (PsA) is a chronic immune-mediated inflammatory disease that affects peripheral joints, entheses and axial sites along with skin and nails. In addition to the characteristic extra-articular features, such as uveitis and inflammatory bowel disease, PsA is also associated with comorbidities [1, 2].

Interplay between the adaptive and innate immune system, activation of T cells and subsequent production of inflammatory cytokines such as TNF, interferon (IFN)-γ, IL-17 and IL-22 have a dominant pathogenic role in psoriatic plaques [3, 4]. In PsA, CD8+ T cells may represent key players that recognise antigens presented by human leukocyte antigen (HLA) class 1 molecules [5]. CD8+ T cells are found in greater numbers in synovial fluid and in entheseal sites while CD4+ T cells are more abundant in synovial tissue [5–7].

Regulatory T cells (Tregs) are a subset of CD4+ CD25high T lymphocytes that require forkhead box transcription factor (FOXP3) for their suppressive function [4, 8, 9]. Tregs derived from the thymus mediate suppression through a contact-dependent mechanism including cytotoxic T-lymphocyte antigen 4 (CTLA-4) [10]. Increased Treg expression compared to normal skin has been reported in psoriatic lesions, irrespective of the disease severity. Previous studies indicated an abnormal phenotype and impaired function of synovial Tregs in rheumatoid arthritis (RA) and increased FOXP3 expression in synovial CD4+ T cells in rheumatoid factor (RF)-negative RA and PsA compared to seropositive RA [10–12]. Tregs isolated from peripheral blood and psoriatic skin have dysfunctional activity in suppressing effector T-cell responses in psoriasis [13–16].

Due to the heterogeneity of PsA, identifying appropriate treatment for each individual can be challenging. Patients with peripheral arthritis that does not respond to disease-modifying antirheumatic drugs (DMARDs) are recommended for treatment with apremilast or biologic agents (TNFi, IL-12/23i and IL17i), IL-12/23i and IL17i) are recommended [17]. Since not all patients respond to these agents and many patients’ initial responses wane over time due to immunogenicity, poor tolerability and/or adverse events, it has been important to develop new therapies that target other key cytokines and immunologic pathways [18]. Targeting T cells, the main mediators of the pathogenic processes in PsA, can potentially have a significant role in the future immunotherapy of the disease.

Abatacept is a soluble, fully human fusion protein consisting of the extracellular domain of CLTA-4 linked to human IgG1 [19]. Abatacept selectively binds to CD80/86 on antigen-presenting cells blocking the costimulatory interaction with CD28 on T-cells, and thereby inhibits optimal T-cell activation and decreases production of inflammatory cytokines such as TNFα, INFγ, IL-2 and IL-6 [20–22]. Abatacept is approved in RA and juvenile idiopathic arthritis [23, 24]. One phase 2 randomised controlled trial of abatacept in PsA showed significant American College of Rheumatologists (ACR)20 responses, but only modest improvement in skin measures compared to placebo (PBO) [25]. More recently, a randomised, placebo-controlled, phase 3 trial of abatacept in PsA reported benefits of abatacept in ACR20 responses, regardless of TNFi exposure, with modest benefit on psoriasis lesions [26].

A few studies have evaluated synovial changes after different therapies in PsA [27], but the effect of abatacept on synovial and skin tissue has not been previously studied. Therefore, our aim was to study changes in immunohistochemical markers of synovial and skin inflammation, including FOXP3 expression, after introducing abatacept treatment in PsA. Secondary objectives were to (1) assess clinical outcomes and magnetic resonance imaging (MRI) changes over time, (2) evaluate the impact of a short period of abatacept 3 mg/kg of body weight treatment compared to PBO and (3) investigate if cell markers of synovial inflammation correlate with disease activity measures and MRI synovitis scores.

## Methods

### Patients

PsA patients fulfilling the classification criteria for psoriatic arthritis (CASPAR) [28] with active disease of ≥3 months duration (tender joint count ≥3 and swollen joint count ≥3), aged 18 to 80 years were recruited. Patients had to have clinical synovitis of the knee and have agreed to undergo synovial biopsies. Presence of a psoriatic skin lesion was not requisite. Participants must not have been exposed to a previous biologic DMARD. Methotrexate (MTX) was the only concomitant synthetic DMARD (sDMARD) permitted in the study. Patients who were on a stable dose of MTX (7.5–25 mg/week) for >3 months prior to randomisation remained on this stable dose for the duration of the study. A decrease in the MTX dosage was allowed in cases of toxicity. The use of a stable dose of prednisolone <10 mg/day or its equivalent, and of a nonsteroidal anti-inflammatory drug (NSAID) was permitted during the study. Decrease in NSAIDs was permitted but only if due to toxicity.

Key exclusion criteria were concurrent autoimmune diseases or skin conditions other than plaque psoriasis, arthritis mutilans, pregnancy, breast feeding, evidence of latent or active tuberculosis, evidence of chronic or clinically significant infection or malignancy.

Informed, written consent was obtained from all study participants according to the Declaration of Helsinki. The study was approved by St. Vincent’s Healthcare Group Ethics and Medical Research Committee.

### Study design and treatment

This study was a single-centre, placebo-controlled, crossover study of 15 patients with PsA. Patients were randomised to receive abatacept 3 mg/kg of body weight (approved dosing for RA) or PBO infusions on day 1, 15 and 29; thereafter abatacept 10 mg/kg of body weight was administered every 28 days for 5 months (on day 57, 85, 113, 141 and 169). Treatments were administered as a 30-minute intravenous infusion; dosing was calculated based on body weight at screening (500 mg, 750 mg and 1000 mg for patients weighing <60 kg, 60–100 kg and >100 kg, respectively).

### Clinical and laboratory assessments

Demographic data collected included age, sex, psoriasis and psoriatic arthritis disease duration, menopausal status, previous use of sDMARDs, current use of MTX, oral corticosteroids and calcium and vitamin D supplements. Clinical assessments were performed at each visit. Clinical variables included disease activity score in 28 joints (DAS28) with four variables (DAS28-erythrocyte sedimentation rate (ESR) and DAS28-C-reactive protein (CRP)), tender joint count (TJC) 28 and 68, swollen joint count (SJC) 28 and 66, number of digits with dactylitis, enthesitis score based on the Leeds enthesitis index (LEI), number of affected nails on the hands (0–10) and psoriasis area severity index (PASI) [29, 30]. Patients’ self-reported parameters such as duration of early morning stiffness (EMS), visual analogue scale for global health (GVAS), pain and fatigue scores, health assessment questionnaire (HAQ), dermatology life quality index (DLQI) and PsA-specific quality of life score (PsAQol) were recorded [30, 31]. Laboratory assessments included a recording of the following parameters: RF positivity, antibodies against cyclic citrullinated peptides (aCCP), ESR and CRP.

### Efficacy and safety assessments

Joint and skin-related response to abatacept or PBO was assessed using the European League Against Rheumatism (EULAR) response criteria and a 50% improvement in the PASI score (PASI50) [32]. Safety evaluations were obtained at each visit including routine laboratory tests and monitoring of adverse events (AEs) for severity of the event (mild, moderate, severe or very severe), the investigator’s opinion of its relationship to abatacept/PBO treatment (not related, unlikely or possibly related) and information on outcomes.

### Synovial and skin biopsies

Arthroscopy of the same involved knee joint was performed using a Wolf (Illinois, USA) 2.7-mm needle arthroscope under local anaesthesia at three time points (day 1, 57 and 169). Macroscopic synovitis and vascularity were scored on a visual analogue scale (VAS) (0–100 mm). Synovial membrane biopsies were obtained from at least six sites within the joint (suprapatellar pouch, medial and lateral gutters, infrapatellar and tibiofemoral regions and cartilage pannus junction near the patellar rim) using 1.5-mm grasping forceps [33, 34].

Under local anaesthesia, patients underwent a 6-mm-punch skin biopsy at three time points (day -28, 54 and 166). Biopsies were taken from the centre of a target psoriasis lesion from the same plaque [15].

### Immunohistochemical analysis

Synovial and skin biopsies were embedded in Tissue-Tek optimal cutting temperature (OCT) compound (Sakura, Zoeterwoude, The Netherlands) and stored at -80 °C. Cryostat sections (7 μm) were mounted on slides coated with 3-aminopropyltriethoxysilane (Sigma-Aldrich St. Louis, MO, USA), air-dried overnight, wrapped in foil and stored at -80 °C. A routine three-stage immunoperoxidase labelling technique was used [35]. Tissue sections were thawed and acetone-fixed for 10 minutes. Immunostaining was performed on the Dako Autostainer 48 Link. Sections were processed using the Dako Envision FLEX Mouse Linker kit according to the manufacturer’s instructions. DAB was used as the chromogen and slides were counterstained with hematoxylin. The synovial and skin sections were incubated with mouse monoclonal antibodies against T-cell-specific markers CD3 (1:25), CD4 (1:20), CD8 (1:50), CD31 (Dako Ready to use Antibody, Clone numbers: CD3 – F7.2.38, CD4 – 4B12, CD8 – C8/144B, CD31 – JC70A) (Dako, Glostrup, Denmark), and FOXP3 (1:500) (Abcam, Cambridge, UK, Clone: 236A/E7). Sections were also incubated with an appropriate isotype-matched mouse monoclonal antibody (IgG1). Negative control slides omitting the secondary antibodies were also stained.

CD3, CD4 and CD8 expression in the biopsies were scored using a well-established semi-quantitative scoring method on a 5-point scale ranging from 0 to 4 (0 = no staining, 1 ≤ 25%, 2 = 25–50%, 3 = 50–75%, 4 ≥ 75% staining). For evaluation of CD4+ cells, only cells with lymphocyte morphology were included as CD4 can be expressed by macrophages [36, 37]. CD31-expressing microvessels were assessed under × 400 magnification by counting the number of vessels staining per five high power fields (HPF) on two levels when available (=10 HPF/slides). The result for one biopsy was extrapolated from two HPFs, due to the size of the tissue sample. Intranuclear staining for FOXP3 expression was assessed in a semi-quantitative manner as a percentage over 300 cells. (More specifically, 300 lymphocytes were counted at objective × 40 in “hot spots”, both in lymphoid aggregates and lymphoid infiltrates, and the number of FOXP3+ cells was divided by 3 to obtain a percentage; the result was then the average rounded up or down to the nearest whole number.)

### Immunofluorescence staining of synovial tissue

To examine synovial tissue co-localization of CD4 with FOXP3, dual immunofluorescence staining was performed on cryostat synovial sections. Cryostat sections were defrosted at room temperature for 20 minutes and fixed in acetone for 10 minutes. Slides were washed in PBS for 5 minutes. Non-specific binding was blocked using 1% casein in PBS for 20 minutes. Slides were co-incubated with rabbit monoclonal antibody against CD4 (1:500, Abcam, Clone: EPR6855) and mouse monoclonal anti-FOXP3 antibody (1:500, Abcam, Clone: 236A/E7). IgG control antibodies were used as negative controls. Following overnight incubation in a humidified chamber at 4 °C, immunofluorescence sections were co-incubated with Alexa 488 goat anti-mouse superclonal™ secondary antibody (1:200, Invitrogen) and Cy^TM^3-conjugated goat anti-rabbit secondary antibody (1:500, Jackson ImmunoResearch) for 60 minutes. Sections were mounted with Antifade (Molecular Probes) and assessed by immunofluorescence microscopy (Olympus BX51). The number of CD4+FOXP3+ Tregs at baseline and 6 months was scored manually by two independent readers on cryostat sections by identifying cells that had clear signals for co-expression of CD4 and FOXP3 on merged images.

### Magnetic resonance imaging

MRI scan of the same involved knee was performed in each patient 3 days prior to arthroscopy at baseline, 2 and 6 months using a 1.5 T Signa Excite HD MRI scanner (GE Healthcare, UK) and a dedicated eight-channel array HD knee surface coil, with patients lying supine. The examinations performed included intravenous contrast (Gadolinium diethylenetriamine penta-acetic acid) enhanced, T1-weighted, fat-suppressed pulse sequences in coronal, sagittal and axial planes. The following scanning parameters were used: coronal, repetition time (TR) 640 ms; echo time (TE) 16; slice thickness 4/1 mm; field of view (FOV) 18; NEX 2; matrix 512 × 256; sagittal, TR 500; TE 16; slice thickness 4/1 mm; FOV 22; NEX 2; matrix 256 × 192 axial, TR 440; TE 11; slice thickness 3/1.5 mm; FOV 16; NEX 3; matrix 224 × 192.

Images were scored using the psoriatic arthritis MRI synovitis score (PsAMRIS) semi-quantitative method by one consultant radiologist with a special interest in musculoskeletal radiology, who was blind to patient identity and scan chronology. Each knee was divided into four anatomical regions ((medial (MED) and lateral (LAT) parapatellar recesses, intercondylar notch (ICN) and suprapatellar pouch (SPP)) and a synovitis score ranging from 0 to 3 was assigned to each region (0 = normal synovium, 1 = diffuse, even thickening, 2 = nodular thickening, 3 = gross, nodular thickening) based on the overall impression of the severity of synovial abnormality in the three orthogonal scanning planes. A total MRI synovitis score (MRIS) from the four regional scores ranging from 0 to 12 was calculated and used for analysis [38, 39].

### Statistical analysis

Statistical analysis was performed using SPSS for Windows version 20.0 (Armonk, NY, USA). Baseline characteristics were described using mean (SD), median (IQR) for continuous variables and percentage for counts. The Wilcoxon test and the paired sample *t* test were used to compare responses to abatacept treatment for clinical measures, synovitis macroscopic scores and immunohistological staining scores at 0 vs 2 months, 2 vs 6 months and 0 vs 6 months. To measure the effect of the first 2 months of abatacept or PBO treatment over time on cell markers of inflammation in the group we used a general linear model (GLM) repeated measure (within-subject factor: 0, 2 and 6-month time points, between-subject factors: abatacept and PBO treatment groups, and treatment duration). Correlation between synovial cell markers, macroscopic scores, clinical parameters and MRI scores were analysed using Pearson correlation. *P* < 0.05 was considered as statistically significant.

## Results

### Demographic and disease-specific characteristics of the patients

**Fig. 1:**
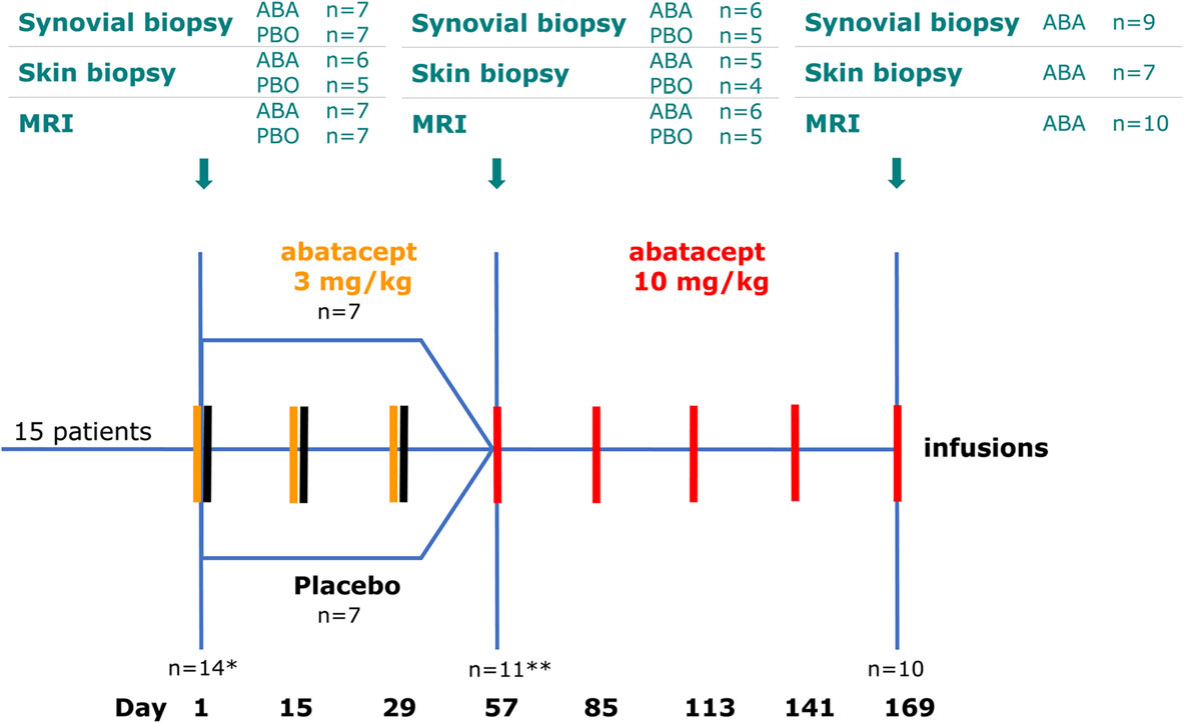
Study design. Numbers of patients who underwent synovial and skin biopsies, and magnetic resonance imaging (*MRI*) in the abatacept (*ABA*) and placebo (*PBO*) study arms at each time point. *One patient was not randomized (screening visit and first MRI only). **Four patients were withdrawn from the study (two patients due to commitment issues (one in the 3 mg/kg of body weight treatment arm and one in the PBO arm)

Fifteen patients (8 female and 7 male) with mean age of 45 (±14.6) years were recruited, and 14 subjects were randomised. Seven patients received abatacept 3 mg/kg of body weight and seven patients were given PBO infusions on day 1, 15 and 29. Eleven patients received he abatacept dose of 10 mg/kg of body weight from day 57. In total 10 patients completed the study (Fig. 1). Out of 14 randomised subjects, 2 patients dropped out due to commitment issues (1 patient in the 3 mg/kg of body weight treatment arm and 1 in the PBO arm) and 2 patients were withdrawn because of an acute disease flare (1 patient in the 10 mg/kg of body weight treatment arm on day 71 and 1 in the PBO arm on day 40). Demographic and disease-specific characteristics of the group are summarised in Table 1. All patients were of Caucasian race. Mean disease duration was 18 years for psoriasis and 10 years for PsA. Four patients were on a stable dose of MTX at the time of recruitment (one patient in the 3 mg/kg of body weight treatment arm and three in the PBO arm) and stayed on the same dose of MTX, the remainder had not received any prior DMARDs; one patient (PBO arm) was on a stable dose of prednisolone 5 mg/day.

**Table 1:** Patients’ demographic and clinical characteristics at baseline

Variable	Value
Demographic parameters	
Age (years)	45 (±14.6)
Female/male, *n* (%)	8 (53)/7 (47)
Age onset of psoriasis (years)	23 (±15)
Psoriasis disease duration (years)	18 (±12)
Age onset of PsA (years)	35 (±15)
PsA disease duration (years)	10 (±9.6)
Previous sDMARDs, *n* (%)	3 (20)
Concomitant sDMARDs (methotrexate), *n* (%)	4 (27)
Currently taken corticosteroids, *n* (%)	1 (7)
Clinical parameters, physician’s assessment	
aCCP+, n (%)	0
RF+, *n* (%)	1 (7)
ESR (mm/h)	27 (±11)
CRP (mg/L)	19 (±10.4)
DAS28-ESR	4.9 (±1)
DAS28-CRP	4.7 (±0.9)
TJC 68	11.5 (±11)
SJC 66	6.9 (±7.4)
Dactylitis (0–20)	1.1 (±1.8)
Enthesitis (0–6)	1.6 (±1.6)
Nail (0–10)	5 (±3.8)
PASI (0–72)	4.5 (±4.6)
Clinical parameters, patient-reported	
Stiffness (minutes)	46 (±35.9)
Pain (0–10)	5.9 (±3.1)
GVAS (0 − 100)	56 (±28.6)
Fatigue (0–10)	5.9 (±2.8)
HAQ (0–3)	0.9 (±0.7)
DLQI (0–30)	4.8 (±7.2)
PsAQol (0–20)	8.6 (±5.7)

Results are presented as mean ± SD, median (IQR) or percentage. Dactylitis, the number of affected digits. Enthesitis score is based on the Leeds enthesitis index. Nail, the number of affected nails on the hands. Stiffness is early morning stiffness. Pain scale ranges from 0 = no pain to 10 = pain as bad as it could be. The visual analogue scale for global health (GVAS) ranges from 0 = worst imaginable health state to 100 = best imaginable health state. The fatigue scale ranges from 0 = no fatigue to 10 = fatigue as bad as it could be. The health assessment questionnaire HAQ scale ranges from 0 = no difficulty to 3 = unable to perform activity. *PsA* psoriatic arthritis, *aCCP* antibodies against cyclic citrullinated peptides, *RF* rheumatoid factor, *sDMARD* synthetic disease-modifying antirheumatic drug, *aCCP* antibodies against cyclic citrullinated peptides, *RF* rheumatiod factor, *ESR* erythrocyte sedimentation rate, *CRP* C-reactive protein, *DAS28*-*ESR*/*CRP* disease activity score based on a 28-joint assessment of pain or swelling using the ESR/CRP-based formula (www.das-score.nl), *TJC* tender joint count, *SJC* swollen joint count, *PASI* psoriasis area and severity index, *DLQI* dermatology life quality index, PsAQol psoriatic arthritis-specific quality of life score

### Clinical responses of arthritis and psoriasis

All patients had active joint disease at baseline as reflected by both physicians’ assessments and patients’ reported outcome measures (PROMs), such as morning stiffness, pain, global health, fatigue, HAQ or PsAQoL scores. Mean TJC68, SJC66, DAS28-ESR and DAS28-CRP were 11.5 (±11), 6.9 (±7.4), 4.9 (±1) and 4.7 (±0.9), respectively. Skin-related measures showed mild to moderate psoriasis with mean PASI, DLQI and nail scores 4.5 (±4.6), 4.8 (±7.2) and 5 (±3.8), respectively (Table 1). DAS28-ESR (*P* = 0.01) and DAS28-CRP (*P* = 0.042) improved as early as 2 months. Significant improvements in most of the joint-related measures and PROMs were observed at 6 months compared to 2 months and to baseline (data not shown). There was very little change in skin-related outcomes. Change in PASI and DLQI from 2 to 6 months was -5.4 (±11.7) and -4.2 (±11.1) in the treatment, and -1.15 (±2.6) and 0.17 (±2.2) in the PBO group, respectively.

According to the EULAR response criteria 73% and 90% of patients were EULAR responders after 2 and 6 months of treatment (46% and 80% good responders, 27% and 10% moderate responders, respectively). PASI50 response at 2 and 6 months was seen in only 9% and 30% of the patients.

### Improvement of knee synovitis on MRI

MRI scans were available at 0, 2 and 6 months for 15, 11 and 10 patients, respectively. MRI synovitis scores improved in each anatomical region of the knee throughout the study. Mean total MRIS at baseline, 2 and 6 months were 8.1 (±3), 7.3 (±3.8) and 6.2 (±4.3), respectively. There was a significant reduction in total MRIS from 2 to 6 months (*P* = 0.047) and from 0 to 6 months (*P* = 0.016). MRIS at 6 months was associated with change in DAS28-CRP from 2 to 6 months (rho = -0.63, *P* = 0.049).

### Macroscopic evaluation of the synovium

Synovial inflammation as assessed by arthroscopy showed significant improvement throughout the study. Mean macroscopic scores at baseline and 2 and 6 months were 73 (±22.1), 41 (±29.7) and 44.4 (±34.3) for synovitis, and 59 (±29.7), 46 (±27.6) and 34 (±29.6) for vascularity, respectively. Significant reduction in synovitis scores was noted from 0 to 2 months and from 0 to 6 months (*P* = 0.016), and in vascularity scores from 0 to 6 months (*P* = 0.039).

### Immunohistological analyses of synovial and skin tissues

**Fig. 2:**
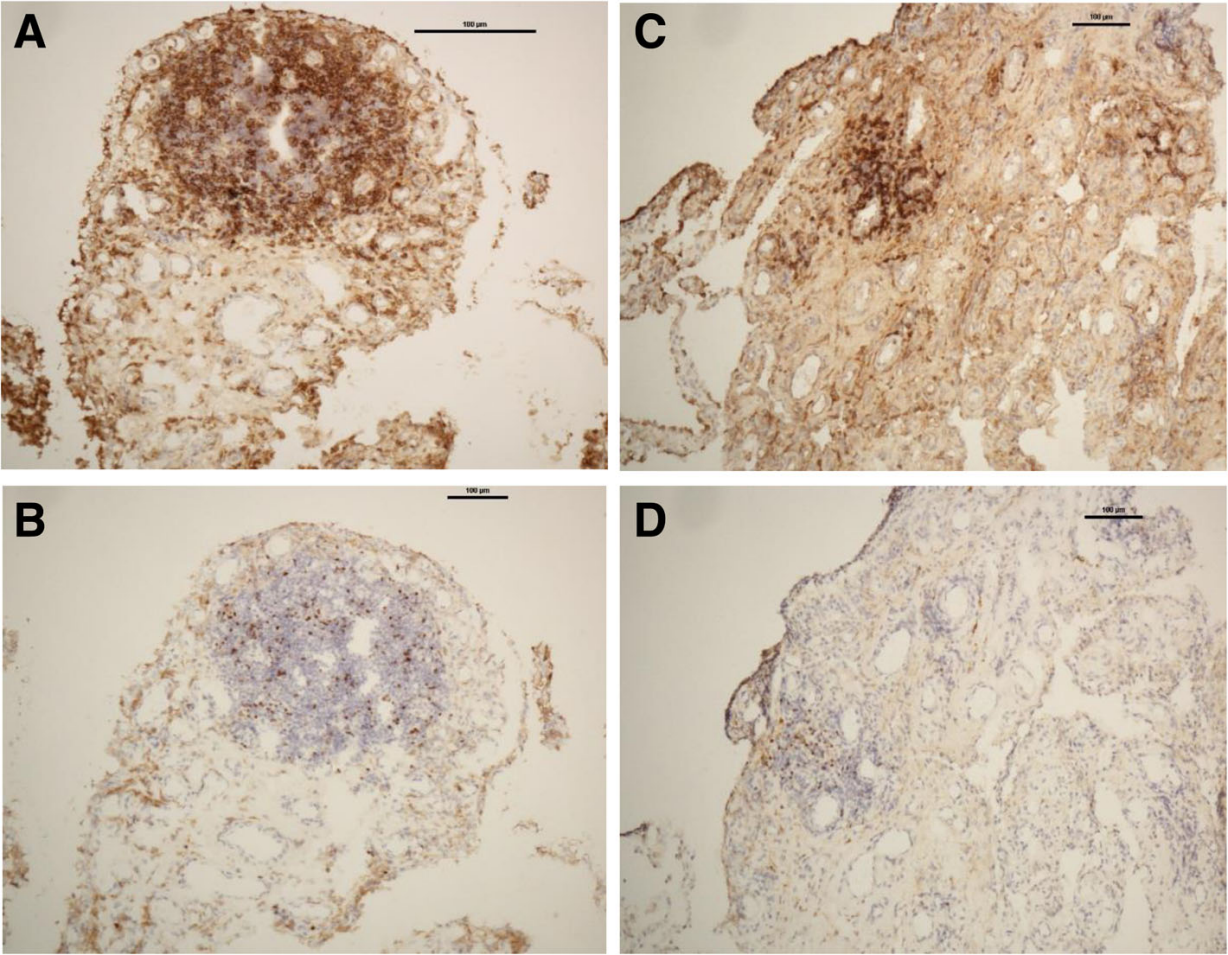
Immunohistological comparison of the expression of CD4+ and forkhead box protein transcription factor 3-positive (FOXP3+) T cells (magnification × 10) in the synovial tissue prior to and at 6 months after abatacept treatment (patient #6). Baseline images show focal infiltration of CD4+ T cells (**a**) and FOXP3+ T cells (**b**) in a villus. Decrease in CD4 expression and significant reduction in the number of FOXP3+ T cells are shown (**c** and **d**, respectively)

Synovial and skin biopsies were available at 0, 2 and 6 months for 14, 11 and 9 patients, and for 11, 9 and 7 patients, respectively. Mean scores of immunohistological expression of CD3, CD4, CD8, FOXP3 and CD31 in the synovium and in the skin prior to and following treatment are summarised in Table 2. There was a trend toward reduction in CD8+ T cells, a reduction in CD4 expression (*P* = 0.073) and a significant reduction in FOXP3 expression (*P* = 0.027) in the synovium following treatment at 6 months. Figure 2 shows representative, single-stained images of decrease in CD4+ cells and in FOXP3+ cells prior to and at 6 months after abatacept treatment. FOXP3 expression was observed mostly in lymphoid follicles; some peri-vascular staining was seen and some lining layer staining was occasionally observed.

**Table 2:** Synovial and skin immunohistological expression of CD3, CD4, CD8, FOXP3 and CD31 prior to and following abatacept treatment

	Baseline		2 Months		6 Months	P value		
						0 vs 2 months		0 vs 6 months
	ABA	PBO	ABA	PBO	ABA	ABA	PBO	ABA
Synovium								
CD3	2.66 (±1.2)	1.8 (±0.8)	2.3 (±1)	2.25 (±1.5)	2.22 (±1.3)	0.679	0.824	0.739
CD4	2.83 (±1.2)	2.4 (±1.5)	2.3 (±0.8)	2.75 (±1.3)	1.78 (±1.1)	0.296	1	0.073
CD8	1.66 (±0.8)	2 (±1)	1.3 (±1)	1.75 (±1)	1.33 (±0.7)	0.576	0.604	0.129
FOXP3	5 (±6)	4.4 (±4.4)	2.8 (±2.2)	4.6 (±4)	1.13 (±2.4)	0.407	0.807	0.027*
CD31	78.3 (±53.9)	85.6 (±50.2)	67.6 (±75.9)	155.5 (±136)	73.22 (±69.9)	0.621	0.482	0.813
Skin								
CD3	2.6 (±0.8)	2.4 (±0.9)	2.25 (±1.5)	2.5 (±2.1)	2.43 (±1.1)	0.718	0.717	0.891
CD4	3.5 (±0.5)	2.8 (±1.1)	2.75 (±0.9)	4 (±0)	3.14 (±0.9)	0.182	1	0.891
CD8	2 (±0.6)	1.75 (±1)	1.5 (±0.6)	2 (±1.4)	1.71 (±0.8)	0.718	0.423	0.577
FOXP3	16 (±9.5)	9.2 (±10.7)	9.5 ±4.5	17.5 ±2.1	13.14 ±8.3	0.743	0.709	0.497
CD31	61.2 (±38)	65.4 (±20.5)	75.5 ±17.1	64.5 ±10.6	54.71 ±17.2	0.788	0.749	0.612

The Wilcoxon test and the paired sample *t* test were used for analysis. Results are presented as mean ± SD

Number of ABA samples at 0, 2 and 6 months: synovial n = 7, n = 6, n = 9; skin n = 6, n = 5, n = 7, respectively. Number of PBO samples at 0 and 2 months: synovial n = 7, n = 5; skin n = 5, n = 4, respectively. Expression of CD3, CD4 and CD8 was scored on a 5-point scale (0–4). CD31 expression was scored by the number of positive vessels/10 high power fields. Forkhead box protein transcription factor 3 (FOXP3) expression was scored as %/300 cells. *ABA* abatacept group, *PBO* placebo group. *Statistically significant

We did not find significant change in synovial CD3, CD8 or CD31 expression during the study period. A trend toward reduction in CD3+, CD4+ and CD8+ T cells was also seen in the lesional skin in particular in the first 2 months; however, significant change in expression was not observed for any of the cell markers over the 6-month treatment period.

### Dual immunofluorescence staining of synovial tissue

Recent studies have proposed that FOXP3 is not only expressed by Tregs, but can be expressed by other types of lymphoid or myeloid cells, and by some non-haematopoietic cells such as epithelial cells [40]. To further specify the phenotype of FOXP3+ cells we performed dual immunofluorescence staining on synovial tissue sections with anti-CD4 and anti-FOXP3 antibodies.

**Fig. 3:**
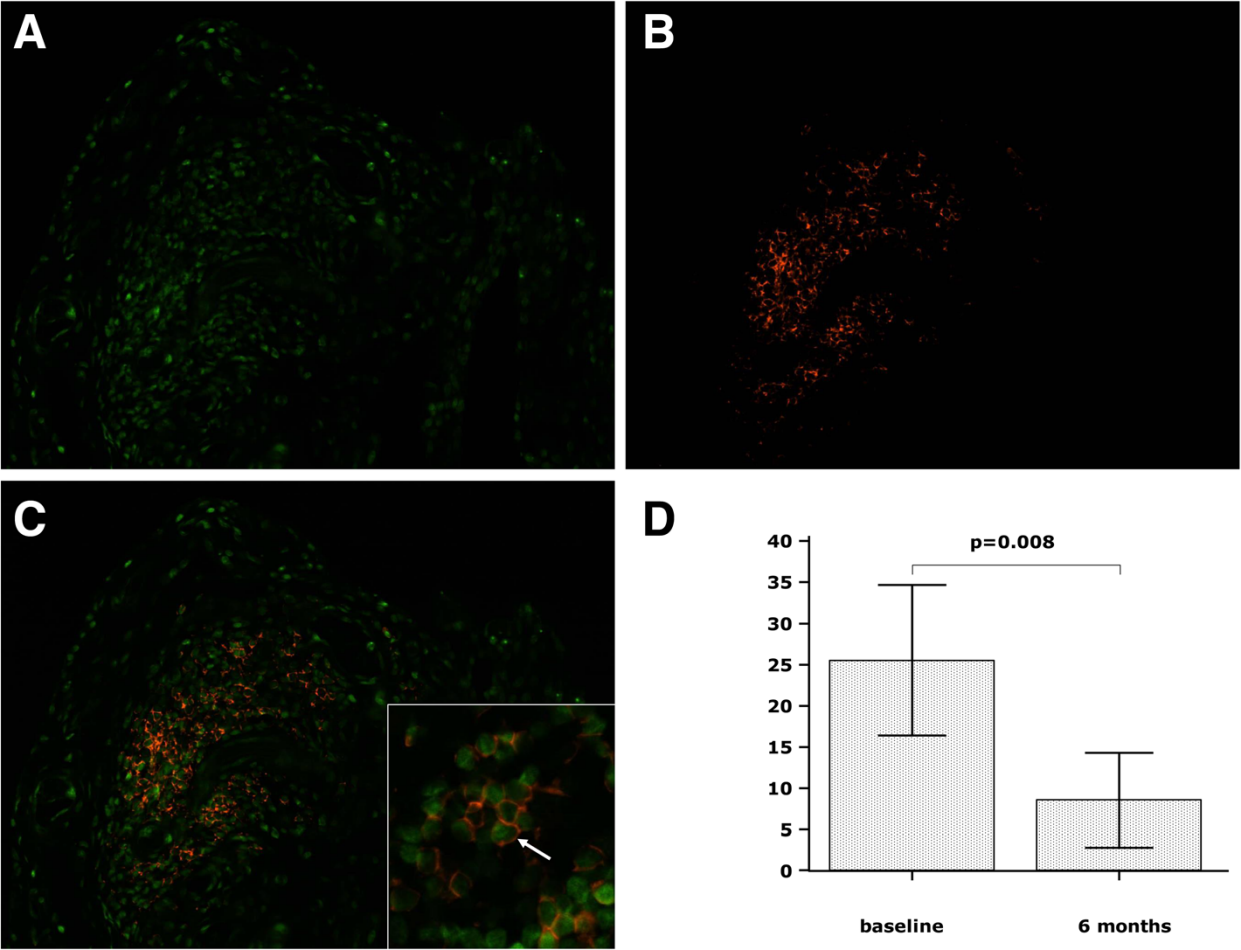
Representative images of immunofluorescent staining in cryostat synovial sections in a patient. **a** Nuclear staining of forkhead box protein transcription factor 3 (FOXP3) (*green*), **b** CD4+ T lymphocytes (*red*), **c** double-stained CD4+FOXP3+ T lymphocytes (*green* nuclear staining surrounded by *red* cytoplasmic staining), insert shows magnification × 40, *white arrow* points to a double-stained CD4+FOXP3+ T cell. **d** Reduction in the number of double-stained CD4+FOXP3+ T cells from baseline to 6 months. Magnification of photomicrographs × 20

Figure 3a-c shows representative images of immunofluorescent staining in synovial tissue (nuclear staining of FOXP3 (green), CD4+ T lymphocytes (red), and double-stained CD4+FOXP3+ T lymphocytes (green nuclear staining surrounded by red cytoplasmic staining). CD4 co-localized with FOXP3 mostly in lymphoid follicles. Figure 3d demonstrates quantification of double-stained CD4+FOXP3+ cells in synovial tissue at baseline and 6- months post treatment. Significant reduction (*P* = 0.008) in the number of double stained CD4+FOXP3+ T-cells was found in patients (n = 9) following 6 months abatacept treatment compared to baseline.

### The impact of a short period of treatment with abatacept 3 mg/kg of body weight or placebo on clinical outcomes and cell markers

Abatacept 3 mg/kg of body weight or PBO was given to 7 patients in each arm on day 1, 15 and 29. Clinical outcomes, PROMs, and cell markers of synovial and skin inflammation were compared between the two treatment arms at 0 and 2 months. Disease activity measures and cell markers or their change over 2 months did not show significant difference between the abatacept and PBO groups (data not shown), with two exceptions: the MRIS was higher at baseline (*P* = 0.02) and FOXP3 expression in the skin was lower at 2 months (*P* = 0.028) in the treatment group compared to the PBO arm. The GLM repeated measure showed that the short PBO treatment did not have a significant effect on the changes in expression of cell markers of synovial and skin inflammation between baseline and 2 and 6 months in the whole group.

### Macroscopic evaluation of synovial inflammation correlates with reduction in synovial cell markers

Both synovitis and vascularity scores correlated with FOXP3 expression at 2 months (rho = 0.7, *P* = 0.024 and rho = 0.65, *P* = 0.041, respectively), and improvement in vascularity score correlated with reduction in FOXP3 expression from 2 to 6 months (rho = 0.64, *P* = 0.034).

### Safety

AEs were reported in a total of 12 patients (86%). Four patients (57%) in the treatment arm receiving abatacept 3 mg/kg of body weight, 6 patients (86%) in the PBO arm, and 8 patients (73%) who received the dose of 10 mg/kg of body weight from day 57 experienced mild or moderate AEs. There were no cases of acute infusion reaction. AEs possibly related to abatacept or PBO as per the investigator’s opinion were reported in one (headache, influenza-like symptoms) and two (diarrhea, flare of psoriasis and arthritis) cases, respectively. There were no serious adverse events.

## Discussion

This single-centre, placebo-controlled, crossover study demonstrates that abatacept reduced the immunohistological expression of CD4+ T cells and specifically reduced FOXP3+ Treg expression in the synovium over 6 months. Abatacept 10 mg/kg treatment significantly improved clinical measures including MRI synovitis scores and arthritis-related PROMs in PsA with little effect on skin-related outcomes.

Clinical efficacy of abatacept has been reported in patients with active RA, including those with an inadequate response to MTX or TNF inhibitors [41], in early type 1 diabetes mellitus, and more recently, in primary Sjögren’s syndrome [21, 42]. Whilst there was no major clinical response to treatment with abatacept 10 mg/kg of body weight in a phase-2 open-label 24-week trial in ankylosing spondylitis [43], Mease et al. reported efficacy of abatacept on joint symptoms in PsA and identified a differential dosage effect of abatacept, with the dose of 10 mg/kg of body weight having more therapeutic effect on arthritis than the 3 mg/kg regimen [25]. In line with this study, we did not notice significant improvement in joint-related outcomes with the dose of 3 mg/kg of body weight, whereas the 10 mg/kg regimen led to significant improvements in most joint-related measures at 6 months. Similarly, MRI synovitis scores improved significantly over time only after administering the dose of 10 mg/kg of body weight. Abatacept has been shown to have some clinical benefit in the treatment of psoriasis [4]. In an open-label phase-1 study of abatacept in psoriasis, 50% or greater improvement of skin involvement was achieved in patients using doses of 25 and 50 mg/kg of body weight, and the same doses correlated best with the reduction of epidermal hyperplasia and the decrease in activated T cells in the skin [44, 45]. We observed very little improvement in the PASI or DLQI over 6 months, but the treatment doses of 3 and 10 mg/kg of body weight may have been too low to be of clinical benefit.

Studies assessing synovial changes after initiating antirheumatic therapy are limited in PsA. From these studies it can be concluded that successful treatment leads to deactivation of endothelium, reduced vascularity and reduction in infiltrating immune cells [27]. Immunpathological changes in the synovium following abatacept treatment in PsA was evaluated in a single case report, and suggested regression of synovial inflammation in a patient refractory to TNF inhibitors [46]. We found significant reduction in macroscopic scores for both synovitis and vascularity after 6 months of abatacept therapy. A trend toward reduction in CD8+ and CD4+ T cells was observed throughout the study, but a statistically significant effect of the treatment was only detected for the reduction in the number of FOXP3+ Tregs in the synovium.

Tregs play a central role in immune tolerance, suppressing not only autoimmune responses but also aberrant or excessive immune responses to non-self-antigens [4] Data on the presence of FOXP3+ Tregs in inflamed synovial tissue are controversial. Enrichment of Tregs in the inflamed joints was reported in a large cohort of patients with chronic inflammatory arthritis, including patients with PsA [47], whilst another study showed only scarce synovial membrane expression of FOXP3+ T cells during relapse in inflammatory arthritis [12]. Deficiencies in Treg function have been also identified in different autoimmune diseases, including RA and psoriasis. Anti-TNF therapy has been shown to induce a potent population of Tregs in patients with RA; however, after cessation of treatment the natural Treg defect persisted [11, 13–15]. It has been suggested that reduced expression and functional abnormalities in Treg-associated CTLA-4 could contribute to abnormal Treg function in RA and may represent a target for therapy [11]. Data for the effects of abatacept on Treg cell function are limited and conflicting. Bonelli et al. showed that abatacept increased the frequency of Treg cells in RA and inhibited their suppressive capacity [48], whilst another study in RA reported enhanced suppressive capacity of Tregs isolated from peripheral blood [49]. Whether the reduction in FOXP3 expression in the synovium we observed after abatacept treatment is protective or pathogenic remains unclear, since our study did not include detailed phenotypic or functional analyses of the Tregs. However, in view of the significant improvement seen in arthritis measures we speculate that the reduction in potentially dysfunctional Tregs may be protective. In line with our observations, a lack of retention of Tregs has been reported after successful anti-inflammatory treatment with intra-articular glucocorticoid injection, suggesting that a specific treatment that modulates local FOXP3+ Treg numbers and function would be of benefit for patients with joint inflammation [12].

A study investigating the role of Tregs in the pathogenesis of psoriasis found increased Treg expression in lesional skin compared to normal skin, in particular in the chronic plaque type of psoriasis, irrespective of disease severity [15]. It has been proposed that the differential dosage effect of abatacept in PsA that was reported in a previous study may be due to the suppression of Tregs at higher doses [19, 25]. In this study, we did not observe a better skin response with low-dose abatacept, possibly due to the small sample size, although the study by Abrams et al. [45] suggests that the abatacept doses used in our study may well have been too low to achieve meaningful skin response.

In contrast to changes seen in the synovial tissue, we did not demonstrate significant immunohistological change in skin involvement in response to abatacept. Different cytokine milieu in the joints compared to the skin may account for this. A recent comparative genomic profiling study of the synovium versus skin lesions in PsA revealed clearly distinct gene expression patterns in the skin compared to the synovium with a stronger IL-17 gene signature in the skin, and more equivalent TNF and IFN-γ gene signatures in both tissues. The authors found fewer Tregs in the synovium compared to the skin; however, the difference was not significant [50].

## Conclusions

In conclusion, this is the first study to assess both synovial and psoriatic skin immunopathological changes following abatacept treatment in PsA. The results of the study suggest that treatment with abatacept 10 mg/kg of body weight is associated with significant improvement in the joint-related clinical outcomes but not skin-related outcomes in biologic DMARD-naïve PsA patients and might be an effective treatment option in patients with PsA. We observed different patterns of efficacy of abatacept in the joints and in lesional skin. Reduction in FOXP3+ T-cell expression in the synovium but not in the psoriatic lesion following abatacept treatment may indicate abnormal Treg function in PsA with differential suppressive capacity in the synovium compared to the skin. Comprehensive studies are required on both the distribution and function of Tregs, together with their complex interactions with the local inflammatory milieu present in the psoriatic skin lesion and inflamed synovium in PsA.

## Abbreviations

aCCP: Antibodies against cyclic citrullinated peptides
AEs: Adverse events
CASPAR: Classification criteria for psoriatic arthritis
CRP: C-reactive protein
CTLA-4: Cytotoxic T-lymphocyte Antigen 4
DAS28: Disease activity score 28
DLQI: Dermatology life quality index
DMARDs: Disease-modifying antirheumatic drugs
EMS: Early morning stiffness
ESR: Erythrocyte sedimentation rate
EULAR: European League Against Rheumatism
FOXP3: Forkhead box transcription factor
GVAS: Visual analogue scale for global health
HAQ: Health assessment questionnaire
HPF: High-power fields
IgG1: Immunoglobulin G1
IL: Interleukin
INFγ: Interferon gamma
JIA: Juvenile idiopathic arthritis
LEI: Leeds enthesitis index
MRI: Magnetic resonance imaging
MRIS: Total MRI synovitis score
MTX: Methotrexate
NSAID: Nonsteroidal anti-inflammatory drug
PASI: Psoriasis Area Severity Index
PBO: Placebo
PBS: Phosphate-buffered saline
PDE4i: Phosphodiesterase 4 inhibitor
PROMs: Patients’ reported outcome measures
PsA: Psoriatic arthritis
PsAQol: PsA-specific quality of life score
RA: Rheumatoid arthritis
RF: Rheumatoid factor
SJC: Swollen joint count
TJC: Tender joint count
TNFi: Tumour necrosis factor alpha inhibitor Tregs, Regulatory T cells
